# Characterizing heterogeneity in emotional and behavioral problems: Latent class analysis with 507,188 children and adolescents and associations with mobile gaming addiction behavior

**DOI:** 10.1017/S0033291725102869

**Published:** 2025-12-29

**Authors:** Zhengge Jin, Xiuzhi Yang, Lixin Hu, Liqing Yao, Wenxin Ge, Jiaqi Chen, Zhuowen Wu, Sichun Lin, Yinhuan Guo, Yajun Chen

**Affiliations:** Department of Maternal and Child Health, School of Public Health, Sun Yat-sen University, Guangzhou 510080, China

**Keywords:** emotional and behavioral problems, internet gaming disorder, latent class analysis, mobile gaming addiction, SDQ

## Abstract

**Background:**

While mobile gaming addiction (MGA) behavior is increasingly prevalent among children and adolescents, the role of specific emotional-behavioral profiles – particularly their latent patterns – in associating with MGA behavior remains poorly understood. This study aimed to examine these associations and age-related variations.

**Methods:**

Data were analyzed from 507,188 participants aged 6–18 years in the Children’s Growth Environment, Lifestyle, and Physical and Mental Health Development Project, conducted in Guangzhou, China, in 2020. Latent class analysis was performed on parent-reported Strengths and Difficulties Questionnaire (SDQ) data to identify subgroups with distinct emotional and behavioral problems. Associations between SDQ dimensions, latent classes, and MGA behavior were examined using logistic regression analysis.

**Results:**

Five latent classes were identified: ‘Low symptom’ (82.2%), ‘Internalizing’ (0.8%), ‘Peer and prosocial issues’ (4.3%), ‘High difficulties’ (5.0%), and ‘Hyperactive’ (7.6%). Compared to the ‘Low symptom’ class, all other latent classes showed significantly higher risks for MGA, with the strongest association observed in the ‘Internalizing’ class (adjusted odds ratio [AOR]: 2.84; 95% confidence interval [95% CI]: 2.67–3.02). Among SDQ subscales, conduct problems presented the highest association (AOR: 2.08; 95% CI: 2.04–2.12), though all SDQ subdimensions were significantly positively correlated with MGA behavior (all *p* < 0.05). Notably, these associations were consistently stronger in adolescents (aged 13–18 years) than in children (aged 6–12 years).

**Conclusions:**

This study identifies specific SDQ-based risk characteristics for MGA behavior, with adolescents (aged 13–18 years) being the most vulnerable. Future longitudinal studies should verify these associations, and clinicians may prioritize early screening for internalizing and conduct-related difficulties.

## Introduction

Amid rapid technological advancements, smartphones have gained widespread popularity and have become an integral component of daily life (Abi-Jaoude, Naylor, & Pignatiello, 2020). However, for children and adolescents, mobile games are increasingly replacing traditional leisure activities and even encroaching on their study time (Lissak, 2018). Designed to trigger dopamine release through incentives and immersive experiences, these games foster excessive phone reliance, which has been recognized as a form of technological addiction (García-Oliva & Piqueras, 2016; Pan, Chiu, & Lin, 2019). The prevalence of mobile gaming among adolescents is substantial; for instance, Sherry, Lucas, Greenberg, and Lachlan (2006) reported that 68% of adolescents engage in mobile gaming as their weekly form of entertainment (Sherry et al., 2006). Similarly, 67.8% of Chinese minors regularly play online games, with mobile games accounting for 62.8%, according to the fifth edition of the national survey on internet usage among minors (China, 2023). This widespread engagement highlights both the allure of mobile games and their potential impact on adolescent behavior and well-being. Recognizing the escalating impact of such behaviors, major mental disorder diagnostic systems, namely the Fifth Edition of the Diagnostic and Statistical Manual of Mental Disorders and the Eleventh Edition of the International Statistical Classification of Diseases and Related Health Problems, have incorporated Internet Gaming Disorder (IGD) in their appendices or formal versions, indicating that this potential disorder warrants further empirical investigation (Laconi, Pires, & Chabrol, 2017; Müller et al., 2022). Similarly, mobile gaming addiction (MGA) behavior also requires closer examination. As a specific manifestation of excessive gaming behavior, MGA behavior – which is often associated with IGD – is linked to adverse psychological and social outcomes, including impaired academic performance, social isolation, and increased risk of anxiety and depression (Lissak, 2018; Wang, Sheng, & Wang, 2019). The portability and social features of mobile games may accelerate the transition from MGA behavior to IGD, thereby exacerbating psychosocial challenges and creating a cycle of dependency. Consequently, identifying modifiable factors during critical developmental stages could inform targeted interventions to mitigate risks and promote healthier developmental trajectories.

In recent years, the prevalence of emotional and behavioral problems (EBPs) (e.g. depressive and anxiety symptoms, hyperactivity/impulsivity, and aggressive behavior) has been high among children and adolescents (Racine et al., 2021; Sayal et al., 2018), and their association with gaming addiction has attracted extensive academic attention (Chang & Bushman, 2019; Müller et al., 2015). According to the Interaction of Person-Affect-Cognition-Execution model, individuals may resort to the Internet as a coping mechanism to address life challenges and seek compensatory satisfaction (Brand et al., 2019). Empirical evidence has further demonstrated that adolescents experiencing negative emotions and exhibiting social deficiencies are more likely to engage in excessive gaming (Li et al., 2019; Paulus, Ohmann, von Gontard, & Popow, 2018; Schettler, Thomasius, & Paschke, 2024). Poor impulse control, increased sensation seeking, and social inhibition may actually be risk factors for developing MGA behavior (Mark, Daria, & Daniel, 2012). However, previous studies have predominantly adopted a variable-centered approach, focusing on relationships between isolated dimensions and gaming addiction. While this paradigm effectively reveals association mechanisms at the variable level, it operates under the assumption of homogeneous groups, potentially obscuring the heterogeneous characteristics of individuals. In reality, EBPs often show multidimensional comorbidity and significant inter-individual heterogeneity (Cummings, Caporino, & Kendall, 2014; Liu et al., 2001; Spechler et al., 2019). In contrast, the person-centered approaches, such as latent class analysis (LCA), capture information at the individual level and identify subgroups with similar trait patterns (Rosato & Baer, 2012).

Against this background, the introduction of the LCA method to explore natural categorization patterns of EBPs from a person-centered perspective can address limitations in traditional research (Bianchi et al., 2017; Lanza, 2016). However, it remains unclear how distinct underlying categories are differentially associated with MGA behavior among children and adolescents. Systematically examining the multidimensional characteristics of EBPs and their potential categorical associations with MGA behavior is crucial for developing targeted intervention strategies. The objectives of this investigation were to: (1) identify groups of individuals endorsing similar patterns of EBPs using LCA applied to the Strengths and Difficulties Questionnaire (SDQ) (Goodman, 1997) and (2) evaluate the dimensions of these problems, latent classes, and their association with MGA behavior. Given that the epidemiology of EBPs and MGA behavior is age-dependent, we further examined whether these associations varied across age groups. We hypothesized that EBPs in children and adolescents can be divided into distinct underlying groups, which are differentially associated with MGA. Furthermore, these associations are expected to vary by age group.

## Methods

### Participants and procedure

In this cross-sectional, observational study, we analyzed data from the children’s growth environment, lifestyle, physical, and mental health development project (COHERENCE) (Bao et al., 2024), an ongoing cohort survey that collects health-related information annually from a large sample of children and adolescents in Guangzhou, China. Since 2016, all students in Guangzhou, encompassing ~1,600 primary and secondary schools across 11 administrative districts (Liwan, Yuexiu, Haizhu, Tianhe, Baiyun, Huadu, Zengcheng, Conghua, Huangpu, Panyu, and Nansha), have been required to register in the Electronic Health Record System annually during September and October. Participants provided demographic information and annually updated data, including medical examination results and questionnaire responses, within the system, where all personal information was handled using strict desensitization protocols. The study design and methods have been previously reported in detail, and informed consent was obtained from all participants and legal guardians (Bao et al., 2024). The study protocol received ethical approval from the Human Studies Committee of Sun Yat-sen University (Approval Number: L2016-010). All research procedures strictly adhered to the Strengthening the Reporting of Observational Studies in Epidemiology guidelines to ensure methodological rigor and transparent reporting.

Our study included 1,201,802 children and adolescents aged 6–18 years from the COHERENCE project conducted during the 2019/20 academic year, with all participants providing data necessary for calculating the SDQ and MGA behavior. We excluded individuals from the 2020 COHERENCE database who had missing SDQ or MGA behavior information. Participants with missing values for variables, such as gender, age, and other relevant factors, were also excluded from the cross-sectional analyses. Finally, to ensure that observed differences between models were not due to selection bias, only participants with complete data for the exposure and outcome variables were included in the statistical analysis (*n* = 507,188; see Supplementary Figure S1 for the sample selection flowchart).

### Measures

#### Sociodemographic data

Demographic variables encompassed participants’ age, gender (male or female), age group (children [6–12 years] vs. adolescents [13–18 years]), a categorization aligned with established developmental stages and prior research (Noubiap et al., 2022), and only-child status (yes or no). To mitigate potential confounding effects between the SDQ and MGA behavior, additional control variables were incorporated. Specifically, these included family socioeconomic status at enrollment, characterized by parents’ education level (categorized as below senior high school, completed senior high school, completed junior college, or completed college or above); household monthly income (<5,000; 5,000–7,999; or ≥8,000 CNY, with an option for refusal to answer); paternal and maternal smoking status (classified as never smokers or former/current smokers); and physical activity (PA) level (low, moderate, or high). The weekly PA levels of participants were evaluated using the International Physical Activity Questionnaire Short Form, the reliability and test–retest validity of which have been previously established in prior research (Craig et al., 2003; Lee, Macfarlane, Lam, & Stewart, 2011). Data on the frequency and duration of walking, moderate-intensity, and vigorous-intensity activities were collected from both participants and their parents/guardians. Total weekly PA volume was calculated in MET-minutes/week (minutes/week × standardized MET values for each activity type). Participants were categorized into three groups based on established criteria: low (<600 MET-min/week), moderate (600–3,000 MET-min/week), and high (≥3,000 MET-min/week) (Craig et al., 2003).

#### Strengths and Difficulties Questionnaire

The parent-reported version of the SDQ (SDQ-P) was employed to assess EBPs over the past 6 months in children and adolescents (Goodman, 1997). The SDQ-P is a brief, validated screening tool comprising 25 items organized into five dimensions: emotional symptoms, conduct problems, hyperactivity/inattention, peer relationship problems, and prosocial behavior. Each subscale is scored from 0 to 10, with higher scores indicating greater difficulties, except for the prosocial behavior subscale, where higher scores reflect positive social behaviors. The SDQ-P has been extensively validated and demonstrates satisfactory psychometric properties, including high internal consistency and validity across diverse populations, thus establishing it as a reliable instrument for this purpose (Goodman, 2001; Goodman, Meltzer, & Bailey, 1998; Lai et al., 2010). Following the classification proposed by Goodman (2001) (Goodman, 2001), the scores for each subscale were categorized into ‘normal’, ‘border’, and ‘abnormal’ bands. In the current study, these dimensional results were further classified into normal (including border) and abnormal groups.

#### MGA behavior

MGA behavior over the past 3 months was evaluated using the Problematic Mobile Gaming Questionnaire Short Form (PMGQ-SF) (see Supplementary Table S1), a validated screening tool aligned with clinical diagnostic criteria (Pan et al., 2019). The PMGQ-SF comprises four items evaluating physical discomfort, impulsive gaming behavior, increased tolerance, and withdrawal symptoms. Participants rated each item on a 4-point Likert scale (1 = *strongly disagree*, 2 = *somewhat disagree*, 3 = *somewhat agree*, 4 = *strongly agree*). Based on established cutoffs (Jin et al., 2025; Pan et al., 2019), participants were classified into the ‘MGA risk group’ (PMGQ-SF score ≥ 10) or the ‘non-risk group’ (PMGQ-SF score < 10). To ensure response accuracy, parents or guardians assisted younger participants (grades 1–3), while older participants (grades 4 and above) completed the questionnaire independently. In this study, the scale exhibited good internal consistency, as evidenced by a Cronbach’s *α* of 0.872.

### Statistical analysis

Descriptive statistics were calculated for all variables, with categorical variables reported as frequencies (*n*) and percentages (%) and continuous variables expressed as means ± standard deviations (SDs). The *χ^2^* test and independent-samples *t*-test were employed to compare MGA behavior across participants with different characteristics.

LCA was performed to identify the optimal number of latent classes based on SDQ response patterns. As a person-centered analytical approach, LCA utilizes binary indicators to identify distinct latent patterns and assigns individuals to specific classes based on their observed responses. This method provides a robust framework for uncovering heterogeneity within the population by grouping individuals with similar behavioral and emotional profiles. The optimal number of classes was determined by evaluating multiple criteria: statistical fit indices (Bayesian Information Criterion [BIC], Akaike Information Criterion [AIC], and entropy), as well as the interpretability and theoretical relevance of the resulting classes (Nylund, Asparoutiov, & Muthen, 2007). LCA was chosen over other clustering methods due to its model-based framework, which enables rigorous evaluation of model fit and provides robust parameter estimates while accounting for measurement errors in the indicators (Magidson & Vermunt, 2002; Miettunen, Nordström, Kaakinen, & Ahmed, 2016). LCA was conducted using Mplus 8.3 version.

Binomial and multinomial logistic regression analyses were performed to examine the associations between SDQ dimensions, latent patterns, and MGA behavior, expressed as crude odds ratios (CORs) and adjusted odds ratios (AORs) with 95% confidence intervals (CIs). We also examined the specificity of these associations by comparing children (aged 6–12 years) and adolescents (aged 13–18 years) using the ratio of two odds ratios (RORs) (Altman & Bland, 2003). Specifically, we tested whether the associations were unique to adolescents, as hypothesized. All statistical analyses were performed using R (version 4.4.2; R Foundation for Statistical Computing, Vienna, Austria), with statistical significance defined as *p* < 0.05 (two-tailed).

## Results

### Characteristics of the study sample

Among the selected 507,188 participants, 53.7% were boys, with a mean age of 10.07 years (SD = 3.01). The sample comprised 76.7% children and 23.3% adolescents. The abnormal rates for emotional symptoms, conduct problems, hyperactivity/inattention, peer relationship problems, and prosocial behavior were 6.7%, 8.9%, 11.4%, 17.6%, and 10.8%, respectively. Overall, 107,536 (21.2%) participants were identified as having MGA behavior. Compared to participants without MGA behavior, those with MGA behavior were more likely to be boys (22.9% vs. 19.3%), adolescents (22.5% vs. 20.8%), and the only child in their family (21.3% vs. 21.2%). Additionally, the prevalence of MGA behavior was significantly higher among participants with lower parental education levels, parental smoking habits, lower household incomes, and low physical activity levels (*P* < 0.001). Furthermore, the prevalence of MGA behavior was highest among those with abnormalities in the various dimensions of the SDQ (*P* < 0.001). Detailed demographic information and descriptive statistics for MGA behavior are presented in Table 1.

**Table 1.** Baseline characteristics of participants by MGA behavior status (2020, *N =* 507,188)

Variables	Total	MGA behavior {score [*n* (%)]}		*P*-value^a^
		No (<10)	Yes (≥10)	
Age, years [mean (SD)]	10.07 (3.01)	10.02 (3.03)	10.26 (2.97)	<0.001
Gender [*n* (%)]				
Boys	2,72,204 (53.7)	2,09,916 (77.1)	62,288 (22.9)	<0.001
Girls	2,34,984 (46.3)	1,89,736 (80.7)	45,248 (19.3)	
Age group [*n* (%)]				
Children (6–12 years)	3,89,125 (76.7)	3,08,211 (79.2)	80,914 (20.8)	<0.001
Adolescents (13–18 years)	1,18,063 (23.3)	91,441 (77.5)	26,622 (22.5)	
Only child [*n* (%)]				
Yes	1,57,057 (31.0)	1,23,605 (78.7)	33,452 (21.3)	0.258
No	3,50,131 (69.0)	2,76,047 (78.8)	74,084 (21.2)	
Paternal education level [*n* (%)]				
Below senior high school	1,35,666 (26.7)	1,05,921 (78.1)	29,745 (21.9)	<0.001
Completed senior high school	1,28,292 (25.3)	1,00,694 (78.5)	27,598 (21.5)	
Completed junior college	98,161 (19.4)	77,363 (78.8)	20,798 (21.2)	
Completed college or above	1,45,069 (28.6)	1,15,674 (79.7)	29,395 (20.3)	
Maternal education level [*n* (%)]				
Below senior high school	1,45,806 (28.7)	1,14,510 (78.5)	31,296 (21.5)	<0.001
Completed senior high school	1,18,888 (23.4)	93,500 (78.6)	25,388 (21.4)	
Completed junior college	1,11,727 (22.0)	87,662 (78.5)	24,065 (21.5)	
Completed college or above	1,30,767 (25.8)	1,03,980 (79.5)	26,787 (20.5)	
Paternal smoking status [*n* (%)]				
Never smokers	2,41,750 (47.7)	1,92,626 (79.7)	49,124 (20.3)	<0.001
Former/current smokers	2,65,438 (52.3)	2,07,026 (78.0)	58,412 (22.0)	
Maternal smoking status [*n* (%)]				
Never smokers	5,01,520 (98.9)	3,95,445 (78.8)	1,06,075 (21.2)	<0.001
Former/current smokers	5,668 (1.1)	4,207 (74.2)	1,461 (25.8)	
Household monthly income [*n* (%)]				
<5,000 CNY	1,82,063 (35.9)	1,41,574 (77.8)	40,489 (22.2)	<0.001
5,000–7,999 CNY	1,08,174 (21.3)	85,159 (78.7)	23,015 (21.3)	
≥8,000 CNY	1,70,794 (33.7)	1,35,887 (79.6)	34,907 (20.4)	
Refused to answer	46,157 (9.1)	37,032 (80.2)	9,125 (19.8)	
PA level [*n* (%)]				
Low level of PA	55,410 (10.9)	42,053 (75.9)	13,357 (24.1)	<0.001
Moderate level of PA	1,88,875 (37.2)	1,47,466 (78.1)	41,409 (21.9)	
High level of PA	2,62,903 (51.8)	2,10,133 (79.9)	52,770 (20.1)	
SDQ dimensions				
Emotional symptoms {score [*n* (%)]}				
Normal/border (0 ~ 4)	4,73,378 (93.3)	3,76,851 (79.6)	96,527 (20.4)	<0.001
Abnormal (5 ~ 10)	33,810 (6.7)	22,801 (67.4)	11,009 (32.6)	
Conduct problems {score [*n* (%)]}				
Normal/border (0 ~ 3)	4,61,851 (91.1)	3,69,991 (80.1)	91,860 (19.9)	<0.001
Abnormal (4 ~ 10)	45,337 (8.9)	29,661 (65.4)	15,676 (34.6)	
Hyperactivity/inattention {score [*n* (%)]}				
Normal/border (0 ~ 6)	4,49,130 (88.6)	3,60,534 (80.3)	88,596 (19.7)	<0.001
Abnormal (7 ~ 10)	58,058 (11.4)	39,118 (67.4)	18,940 (32.6)	
Peer relationship problems {score [*n* (%)]}				
Normal/border (0 ~ 3)	4,17,809 (82.4)	3,33,884 (79.9)	83,925 (20.1)	<0.001
Abnormal (4 ~ 10)	89,379 (17.6)	65,768 (73.6)	23,611 (26.4)	
Prosocial behavior {score [*n* (%)]}				
Normal/border (5 ~ 10)	4,52,584 (89.2)	3,61,271 (79.8)	91,313 (20.2)	<0.001
Abnormal (0 ~ 4)	54,604 (10.8)	38,381 (70.3)	16,223 (29.7)	

*Note:* Dependent continuous variables were presented as mean (SD), and categorical variables were presented as *n* (%).

*Abbreviations:* CNY, China Yuan; MGA, mobile gaming addiction; PA, physical activity; SD, standard deviation; SDQ, Strengths and Difficulties Questionnaire.

a The *P*-values were calculated using the two independent samples *t*-test for continuous variables or the chi-square test for categorical variables to assess disparities in MGA among children and adolescents.

### Patterns of SDQ

**Figure 1.**
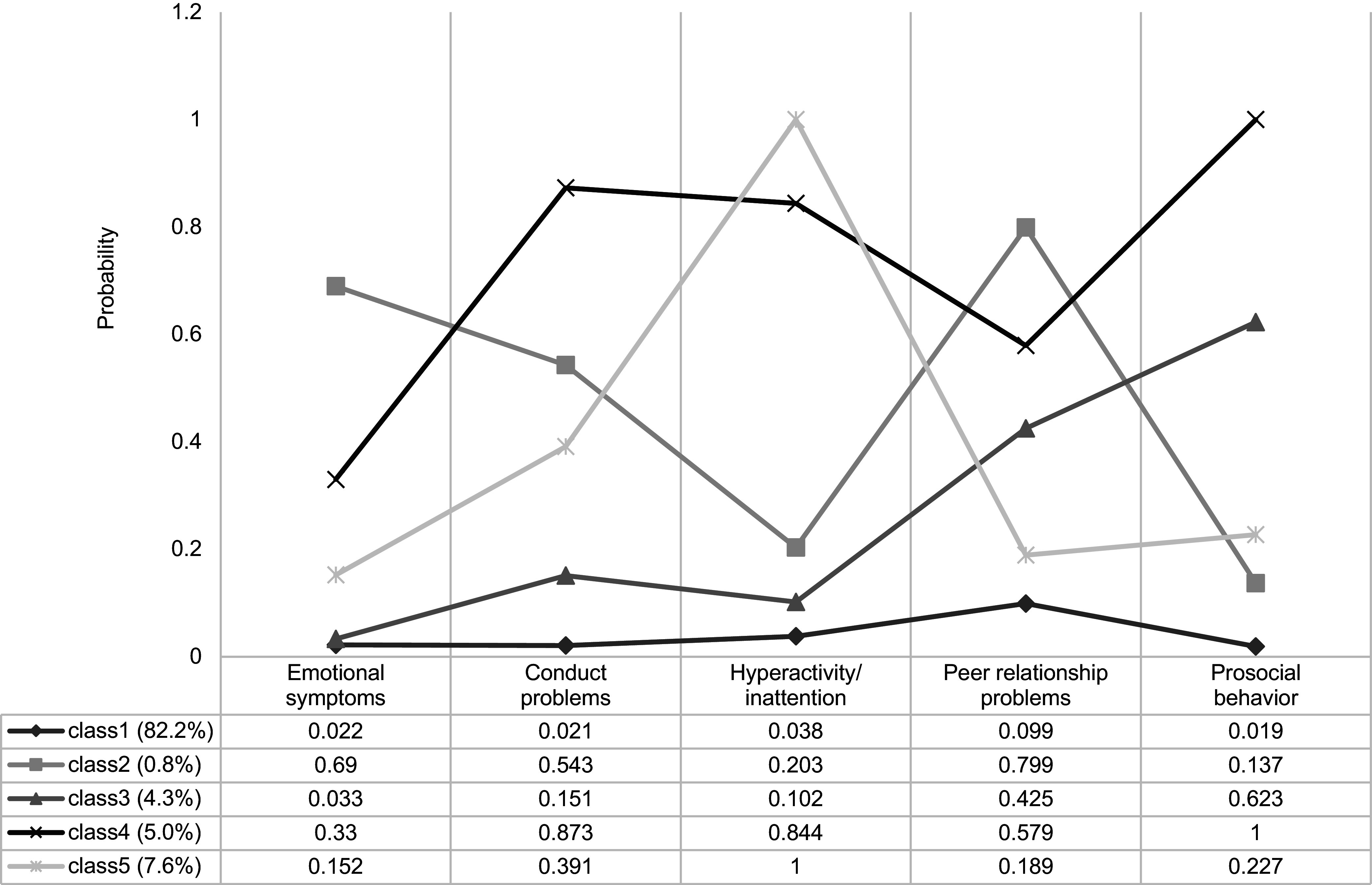
Estimated indicator probabilities and latent classes for all five dimensions of SDQ in the total sample (*N* = 507,188). *Note:* These probabilities correspond to the dichotomized response scale (0 = *Normal/Borderline*; 1 = *Abnormal*), where higher values indicate a greater likelihood of exhibiting problems in each respective dimension. *Abbreviations:* Class 1, Low symptom; Class 2, Internalizing; Class 3, Peer and prosocial issues; Class 4, High difficulties; Class 5, Hyperactive; SDQ, Strengths and Difficulties Questionnaire.

The five-class model was selected as the optimal choice based on statistical fit indices (e.g. AIC and BIC) and interpretability of the latent classes (see Supplementary Table S2). The five retained classes are illustrated in Figure 1. Each class was labeled according to the predominant EBPs that distinguished it from the others. Class 1 (Low symptom, 416,718 participants [82.2% of the sample]) exhibited very low levels of problem behaviors across all domains. Class 2 (Internalizing, 4,250 participants [0.8%]) showed high probabilities of emotional symptoms and peer problems but low to moderate probabilities of conduct problems and hyperactivity/inattention. Class 3 (Peer and prosocial issues, 22,062 participants [4.3%]) was characterized by high probabilities of peer problems and prosocial abnormality. Class 4 (High difficulties, 25,500 participants [5.0%]) displayed moderate to high probabilities across all behaviors, including emotional, conduct, hyperactivity/inattention, peer, and prosocial problems. Finally, Class 5 (Hyperactive, 38,658 participants [7.6%]) demonstrated a very high probability of hyperactivity/inattention but low to moderate probabilities of emotional, conduct, peer, and prosocial problems. Each latent pattern was similarly distributed across both children and adolescents (see Supplementary Figures S2 and S3).

### Association of SDQ dimensions and latent patterns with MGA behavior in children and adolescents

As illustrated in Table 2, all SDQ subdimensions and latent patterns demonstrated significant positive associations with MGA behavior in the total sample (*p* < 0.001). After adjusting for confounding variables, conduct problems exhibited the strongest association with MGA behavior (AOR: 2.08; 95% CI: 2.04–2.12). Compared to Class 1 (Low symptom), all other latent patterns were associated with a significantly higher risk of MGA behavior. Notably, the strongest association was observed in Class 2 (Internalizing) (AOR: 2.84; 95% CI: 2.67–3.02), followed by Class 4 (High difficulties) (AOR: 2.21; 95% CI: 2.15–2.27) and Class 3 (Peer and prosocial issues) (AOR: 2.02; 95% CI: 1.96–2.08). Class 5 (Hyperactive) showed the weakest association (AOR: 1.49; 95% CI: 1.45–1.52).

**Table 2.** Associations of SDQ dimensions and latent patterns with MGA behavior in children and adolescents

Categories	Total			
	COR (95% CI)	*P*-value	AOR (95% CI)^a^	*P*-value
SDQ dimensions				
Emotional symptoms	1.89 (1.84–1.93)	<0.001	1.89 (1.84–1.93)	<0.001
Conduct problems	2.13 (2.09–2.17)	<0.001	2.08 (2.04–2.12)	<0.001
Hyperactivity**/**inattention	1.97 (1.93–2.01)	<0.001	1.96 (1.93–2.00)	<0.001
Peer relationship problems	1.43 (1.41–1.45)	<0.001	1.38 (1.36–1.41)	<0.001
Prosocial behavior	1.67 (1.64–1.71)	<0.001	1.61 (1.58–1.64)	<0.001
SDQ latent patterns				
Class 1 (Low symptom)	1		1	
Class 2 (Internalizing)	2.96 (2.78–3.15)	<0.001	2.84 (2.67–3.02)	<0.001
Class 3 (Peer and prosocial issues)	2.06 (2.01–2.13)	<0.001	2.02 (1.96–2.08)	<0.001
Class 4 (High difficulties)	2.25 (2.19–2.32)	<0.001	2.21 (2.15–2.27)	<0.001
Class 5 (Hyperactive)	1.54 (1.51–1.58)	<0.001	1.49 (1.45–1.52)	<0.001

*Abbreviations:* AOR, adjusted odd ratio; CI, confidence interval; CNY, China Yuan; COR, crude odds ratio; MGA, mobile gaming addiction; PA, physical activity; SDQ, Strengths and Difficulties Questionnaire.

a Adjusted for age, gender, age group, only child, parents’ education level, parents’ smoking status, household monthly income (CNY), and PA level.

### Age difference of SDQ dimensions and latent patterns with MGA behavior in children and adolescents

Table 3 presents the associations between SDQ subdimensions, latent patterns, and MGA behavior stratified by age group (children: 6–12 years; adolescents: 13–18 years). All measures were significantly associated with MGA behavior in both groups. Among children, conduct problems showed the strongest association (AOR: 2.02; 95% CI: 1.97–2.06, *p* < 0.001), whereas hyperactivity/inattention was most strongly associated in adolescents (AOR: 2.61; 95% CI: 2.48–2.76, *p* < 0.001). Compared to Class 1 (Low symptom), Class 4 (High difficulties) had the strongest association in both children (AOR: 2.68; 95% CI: 2.51–2.87, *p* < 0.001) and adolescents (AOR: 3.51; 95% CI: 3.10–3.97, *p* < 0.001). Moreover, RORs indicated that these associations between SDQ dimensions, latent patterns, and MGA behavior were consistently and significantly stronger in adolescents than in children (*p* < 0.05).

**Table 3.** Age-specific associations of SDQ dimensions and latent patterns with MGA behavior in children and adolescents

Categories	Children (6–12 years)			Adolescents (13–18 years)			Ratio of two odds ratios in children versus adolescents	
	*n* (%)	AOR (95% CI)^a^	*P*-value	*n* (%)	AOR (95% CI)^a^	*P*-value	ROR (95% CI)^b^	*P*-value
SDQ dimensions								
Emotional symptoms	7,965 (31.9)	1.86 (1.81–1.92)	<0.001	3,044 (34.3)	1.99 (1.90–2.09)	<0.001	0.93 (0.88–0.99)	0.014
Conduct problems	12,530 (33.6)	2.02 (1.97–2.06)	<0.001	3,146 (39.3)	2.31 (2.20–2.42)	<0.001	0.87 (0.83–0.92)	<0.001
Hyperactivity**/**inattention	16,322 (31.4)	1.88 (1.85–1.92)	<0.001	2,618 (43.0)	2.61 (2.48–2.76)	<0.001	0.72 (0.68–0.76)	<0.001
Peer relationship problems	17,283 (25.6)	1.35 (1.32–1.37)	<0.001	6,328 (28.9)	1.49 (1.44–1.54)	<0.001	0.91 (0.87–0.94)	<0.001
Prosocial behavior	12,005 (28.8)	1.57 (1.53–1.60)	<0.001	4,218 (32.8)	1.74 (1.67–1.81)	<0.001	0.90 (0.86–0.94)	<0.001
SDQ latent patterns								
Class 1 (Low symptom)	59,459 (18.7)	1		19,293 (19.9)	1		1	
Class 2 (Internalizing)	5,224 (31.8)	1.97 (1.91–2.04)	<0.001	1,691 (35.2)	2.20 (2.07–2.34)	<0.001	0.90 (0.84–0.96)	0.002
Class 3 (Peer and prosocial issues)	7,184 (25.4)	1.43 (1.39–1.47)	<0.001	3,630 (31.0)	1.75 (1.67–1.82)	<0.001	0.82 (0.78–0.86)	<0.001
Class 4 (High difficulties)	1,428 (39.6)	2.68 (2.51–2.87)	<0.001	507 (48.5)	3.51 (3.10–3.97)	<0.001	0.76 (0.66–0.88)	<0.001
Class 5 (Hyperactive)	7,619 (33.5)	2.11 (2.05–2.17)	<0.001	1,501 (41.1)	2.66 (2.48–2.85)	<0.001	0.79 (0.74–0.85)	<0.001

*Abbreviations:* AOR, adjusted odd ratio; CI, confidence interval; CNY, China Yuan; MGA, mobile gaming addiction; PA, physical activity; SDQ, Strengths and Difficulties Questionnaire.

a Adjusted for age, gender, only child, parents’ education level, parents’ smoking status, household monthly income (CNY), and PA level.

b Age level differences in the associations were examined via two odds ratios (RORs).

## Discussion

The hypotheses were empirically validated in this study, which, to our knowledge, represents the first investigation into the prevalence and consequences of EBPs, as measured by the SDQ, on MGA behavior in a large group of Chinese children and adolescents. Our key findings indicated that both SDQ subscales and latent patterns were significantly associated with MGA behavior, with conduct problems and ‘Internalizing’ patterns demonstrating the strongest associations. Notably, these associations were significantly more pronounced in adolescents (aged 13–18 years) than in children (aged 6–12 years).

Our study revealed a significant positive association between self-reported exposure to EBPs and an increased likelihood of experiencing MGA behavior, suggesting that psychological distress may serve as a precursor to the development of MGA behavior (Gao, Wang, & Dong, 2022). At the dimension level, externalizing symptoms (e.g. conduct problems and hyperactivity/inattention) showed stronger associations with MGA behavior than internalizing symptoms, potentially reflecting their direct driving effect on addictive behaviors. Given limited direct evidence on MGA behavior, existing frameworks from internet addiction research provide a theoretically sound paradigm for comprehending the relationship between externalizing symptoms and MGA behavior. For instance, a systematic review and meta-analysis on internet addiction have shown that individuals with attention-deficit/hyperactivity disorder, a condition often characterized by hyperactivity and impulsivity, are more susceptible to video game addiction (Wang et al., 2017). Similarly, low self-control and aggressive behaviors have been represented as risk factors for gaming-related problems among children and adolescents (Gentile et al., 2011; Jeong et al., 2020). These findings align with the current study’s results, reinforcing the connection between externalizing symptoms and MGA behavior. The underlying mechanism for the association may lie in the rewarding nature of gaming; those with conduct disorder who overemphasize reward cues due to high sensitivity to rewards and punishment seek instant gratification and high stimulation in games offering instant feedback. Moreover, gaming offers an outlet for pent-up energy and aggression (Dong, Hu, & Lin, 2013; Sethi et al., 2018). For individuals with conduct problems, the virtual world provides a consequence-free space to act out (Wang, Yao, et al., 2017).

Although associations within single dimensions provided a foundation for comprehending the relationship between psychobehavioral issues and MGA behavior, individual psychological characteristics generally manifest in combinatorial patterns. Consistent with prior research on children and adolescents (Kawasaki et al., 2025; Ling, Huebner, He, & Zhong, 2016; Morales et al., 2021; Spechler et al., 2019), this study further probed into the synergy among SDQ dimensions via LCA to identify heterogeneous subgroups that share similar psychological and behavioral traits. We found evidence of several distinct patterns of EBPs in a sample of 507,188 participants. Results from the LCA supported a five-class model consisting of a ‘Low symptom’ class, an ‘Internalizing’ class, a ‘Peer and prosocial issues’ class, a ‘High difficulties’ class, and a ‘Hyperactive’ class. The ‘Low symptom’ group was the most prevalent (82.2%) in the sample, aligning with findings from prior studies (Ling et al., 2016; Spechler et al., 2019). Compared to the three-category model derived from previous SDQ studies that did not consider the prosocial behavioral abnormality dimension (Li et al., 2022; Ling et al., 2016), the results of the LCA in the present study supported a five-category model that offered a more nuanced categorization. The five-category model provides greater nuance than the three-category frameworks derived from earlier SDQ studies (Li et al., 2022; Ling et al., 2016), which omitted the prosocial behavior dimension.

Interestingly, the LCA showed results that differed from those of the individual SDQ subdimensions in relation to MGA behavior. Specifically, compared to the ‘Low symptom’ class, all other classes were associated with an increased risk of MGA behavior, with the ‘Internalizing class’ demonstrating the strongest association with MGA behavior. This discrepancy can be attributed to the following reasons: (1) the co-occurrence of multiple internalizing symptoms generates a cumulative risk that single-dimension analysis fails to capture; (2) there exist interactions between internalizing symptoms and other subclinical features, enhancing the vulnerability to MGA behavior; and (3) the person-centered approach captures qualitatively distinct risk profiles that transcend the simple additive effects of individual symptoms (Goodman et al., 2003; Morales et al., 2021). This is consistent with previous findings emphasizing that emotional and peer interaction problems in children and adolescents may be markers or risk factors for gaming addiction (Burleigh et al., 2019; Jeong et al., 2021; Wang, Zhao et al., 2017; Zhao, Zhou, Wei, & Vogel, 2024). The compensatory satisfaction theory offers a plausible explanation: adolescents with internalizing symptoms (e.g. depression and social anxiety) may use gaming as a maladaptive coping mechanism to address offline emotional deficits or failed social interactions (Liu, Fang, Wan, & Zhou, 2016). Neurally, internalizing symptoms induce alterations in the neurocircuitry associated with addiction-related compulsivity, manifested as prefrontal inhibitory deficits, ventral striatal reward hypersensitivity, and amygdala-prefrontal dysregulation, which collectively drive compulsivity, impaired control, and maladaptive emotion regulation (Jaworska et al., 2020; Wu et al., 2022). Meanwhile, the complexity and diversity of social environment factors (e.g. social skills and family and school atmosphere) and psychological factors (e.g. personality and coping) also play an important role (Brand et al., 2019).

As we expected, adolescents aged 13–18 years with abnormal levels and four latent patterns of EBPs were more likely to exhibit excessive MGA behavior compared with children aged 6–12 years, thereby supporting the third hypothesis. The age-specific strengthening of all associations highlights adolescence as a critical vulnerability window (Crone & Konijn, 2018; Fuhrmann, Knoll, & Blakemore, 2015). Possibly, the late maturation of the dorsolateral prefrontal cortex, combined with heightened emotional reactivity, impairs adolescents’ ability to regulate emotions and control impulses (Crone & Konijn, 2018; Pfeifer et al., 2011). As a result, they are more likely to be influenced by EBPs and develop MGA behavior. Adolescents, facing more significant academic pressures than children, such as competitive entrance examinations and more challenging courses, increasing their risk of emotional and behavioral difficulties like anxiety and depression (Fu, Ren, & Liang, 2022). They are likely to engage in MGA behavior as a means of relaxation and entertainment to reduce stress and escape from real problems. Furthermore, younger children are typically subject to stricter parental oversight, whereas adolescents increasingly exercise autonomy in digital devices and cultivate intricate online social networks. This heightened independence renders adolescents more vulnerable to MGA behavior, particularly when confronting emotional-behavioral challenges (Padilla-Walker et al., 2012). These findings emphasize the necessity of implementing targeted early-intervention strategies in public health to mitigate psychological risks and addictive behaviors among adolescents.

### Strengths and limitations

The current study makes important contributions in several aspects. First, the large size of the sample enables rigorous subgroup analyses with adequate statistical power, enhancing the reliability and generalizability of results. Second, our study showcases the effectiveness of LCA in elucidating the diversity of internalizing and externalizing symptoms among children and adolescents, while also delineating subgroup-specific characteristics. Finally, this research is the first to apply LCA to the association between the SDQ and MGA behavior. Latent classes have stronger predictive validity than single dimensions, enriching the research perspective. The findings offer valuable insights for parents and professionals to develop age-specific prevention and intervention strategies, holding significant practical importance.

However, this study has several limitations. First, causal inferences could not be drawn due to the cross-sectional nature of the present investigation. For instance, while EBPs may contribute to MGA behavior, excessive gaming could also exacerbate mental health difficulties (Wang et al., 2019), highlighting the need for longitudinal follow-up of participants to clarify these complex and potentially bidirectional relationships over time. Next, reliance on self-reported questionnaires from participants and their parents/guardians introduces potential response biases and diagnostic constraints, as objective assessments were lacking. To tackle these issues, digital phenotyping using mobile and wearable devices offers a promising alternative for future research (Sequeira et al., 2019; Walsh et al., 2024). It can be employed for real-time monitoring and detection of changes relevant to EBPs, facilitating more objective screening. Such methods could also supply multidimensional objective data, such as game usage, physiological indicators, and social interaction metrics, to comprehensively explore the relationships between relevant variables. Finally, since data were collected solely in Guangzhou, the findings may not generalize to other regions in China. Multicenter studies conducted across diverse geographical areas would enhance the representativeness and reliability of the results.

### Implications

The paradoxical results provide valuable insights for future research and practical interventions. In future research, multiple analytical methods should be used to comprehensively and deeply explore the relationship between EBPs and MGA behavior among children and adolescents. Simultaneously, more research is warranted to examine the impact of different symptom combinations within latent categories on addiction, aiming to gain a better understanding of the underlying factors of addiction. Regarding practical interventions, family interventions are crucial in preventing MGA behavior among children with EBPs. Parents should shift from ‘intrusive control’ to ‘moderate responsiveness’, by not overly emphasizing schoolwork and permitting children to express negative emotions. Implementing supportive emotion socialization behaviors, including accepting, validating, and encouraging children to express their emotions, empathizing with them, and labeling emotions, as well as modeling healthy emotional expression and regulation (Hajal & Paley, 2020; Morris, Criss, Silk, & Houltberg, 2017). A positive family atmosphere helps children learn to manage their emotions and curtail their dependence on mobile games. In addition, individuals who tend to use the MGA behavior to relieve negative emotions can be offered support in coping with these emotions through structured face-to-face social activities organized by schools and communities (Wendel et al., 2024), which also foster authentic interpersonal interactions among students, reducing excessive reliance on virtual environments. Policy-level measures are equally critical. Governments should regulate online gaming marketing, enforce age restrictions, and restrict the addictive design of game companies to protect children from developing gaming addictions. In 2021, China imposed a policy to reduce adolescent online gaming by limiting playtime to 1 h on weekends and holidays (Xiao, 2021). Although these draconian policy measures appear effective superficially (Colder Carras et al., 2021), they have inadvertently increased short-video consumption (Zhou et al., 2024). In this context, collaborating with parents, schools, and the broader society to form a united force is most likely to promote the development and implementation of successful prevention interventions and policies. Understanding age-related differences helps determine optimal timing for clinical interventions, such as psychosocial education (e.g. emotional regulation training and healthy digital habits) for at-risk children and adolescents with EBPs. Implementing such strategies before the peak vulnerability period in adolescence could maximize their impact on reducing the risk of MGA behavior.

## Conclusions

By integrating dimensional and person-centered approaches, this study establishes a developmentally sensitive framework for stratifying the risk of MGA behavior in Chinese youth. We demonstrate that both dimensions of EBPs (particularly conduct problems) and latent psychosocial patterns (notably ‘Internalizing’ patterns) are associated with increased susceptibility to MGA behavior. These associations are significantly stronger during adolescence, underscoring the critical window of neurodevelopmental plasticity around puberty. Early identification of internalizing profiles could disrupt the progression from recreational gaming to addiction.

## Supporting information

10.1017/S0033291725102869.sm001 Jin et al. supplementary material Jin et al. supplementary material

## Data Availability

The survey is not publicly available, and participants were protected under a certificate of confidentiality issued by the Government of Guangzhou due to the sensitive nature of data collected from all student groups in Guangzhou city. Requests to assess the dataset from qualified researchers trained in human participant confidentiality protocols may be sent to the School of Public Health, Medical College of Sun Yat-Sen University at chenyj68@mail.sysu.edu.cn.

## Supplementary material

The supplementary material for this article can be found at http://doi.org/10.1017/S0033291725102869.
