# Supplementary material for: Characterizing heterogeneity in emotional and behavioral problems: Latent class analysis with 507,188 children and adolescents and associations with mobile gaming addiction behavior
Source: Psychol Med. 2025 Dec 29;55:e389. doi: 10.1017/S0033291725102869 (PMC13058644; doi:10.1017/S0033291725102869)
Supplement: Jin et al. supplementary material [file S0033291725102869sup001.docx]

Supplementary materials for manuscript

**Characterising heterogeneity in emotional and behavioral problems: latent class analysis with 507188 children and adolescents and associations with risk for mobile gaming addiction**

Contents:

1. Supplementary Figure S1. Flowchart of participant selection.

2. Supplementary Table S1. PMGQ-SF items and participants’ response, *N* = 507,188.

3. Supplementary Table S2. Indicators of fit for models with one through seven latent classes

4. Supplementary Figure S2. Estimated indicator probabilities and latent classes for all five dimensions of SDQ in children (*N* = 389,125).

5. Supplementary Figure S3. Estimated indicator probabilities and latent classes for all five dimensions of SDQ in adolescents (*N* = 118,063).

Children and adolescents aged 6–18 years from the COHERENCE project conducted during the 2019/20 academic year were selected (*N*=1,201,802 )

**Final participants (*N*=507,188)**

Remaining participants (*N*=1,200,348)

Remaining participants (*N*=527,069)

Remaining participants (*N*=507,468)

Excluded participants without MGA behaviour data (*N*=14,540)

Excluded participants without SDQ data (*N*=673,279)

Excluded participants without covariates data (*N*=19,601)

Excluded participants with PA data anomaly (*N*=280)

Children (6–12 years )

Adolescents (13–18 years)

**Supplementary Figure S1.** Flowchart of participant selection.

*Abbreviations:* COHERENCE, Children’s Growth Environment, Lifestyle, and Physical and Mental Health Development Project; MGA, Mobile gaming addiction; PA, Physical activity; SDQ, Strengths and Difficulties Questionnaire.

**Supplementary Table S1. PMGQ-SF items and participants’ response, *N* = 507,188.**

| **Items** | **Strongly disagree, *n* (%)** | **Somewhat disagree, *n* (%)** | **Somewhat agree, *n* (%)** | **Strongly agree, *n* (%)** |
| --- | --- | --- | --- | --- |
| 1. I have often experienced dry/sore eyes, muscle aches, or other physical discomforts from playing mobile games for a long duration. | 168,357 (33.2) | 243,703 (48.0) | 88,539 (17.5) | 6,589 (1.3) |
| 2. I often do not plan on playing mobile games, but cannot resist picking up my phone to play (swipe). | 120,792 (23.8) | 205,596 (40.5) | 169,549 (33.4) | 11,251 (2.2) |
| 3. Compared to 3 months ago, I am averaging more time every week playing mobile games. | 164,986 (32.5) | 256,439 (50.6) | 79,114 (15.6) | 6,649 (1.3) |
| 4. If I cannot play a mobile game, I feel restless and irritable. | 194,964 (38.4) | 254,880 (50.3) | 52,185 (10.3) | 5,159 (1.0) |
| *Note:* PMGQ-SF, Problematic Mobile Gaming Questionnaire Short Form. | | | | |

| **Supplementary Table S2.** Indicators of fit for models with one through seven latent classes | | | | | | | |
| --- | --- | --- | --- | --- | --- | --- | --- |
| **Model** | **Number of Free Parameters** | **AIC** | **BIC** | **ssaBIC** | **Entropy** | **LMR** | **BLRT** |
| 1-class | 5 | 1733593.834 | 1733649.517 | 1733633.627 |  |  |  |
| 2-class | 11 | 1618003.745 | 1618126.248 | 1618091.289 | 0.686 | 0.3333 | <0.001 |
| 3-class | 17 | 1610580.945 | 1610770.268 | 1610716.241 | 0.819 | <0.001 | <0.001 |
| 4-class | 23 | 1604060.069 | 1604316.212 | 1604243.117 | 0.762 | <0.001 | <0.001 |
| **5-class** | **29** | **1603076.768** | **1603399.731** | **1603307.567** | **0.807** | **<0.001** | **<0.001** |
| 6-class | 35 | 1602809.004 | 1603198.787 | 1603087.555 | 0.785 | <0.001 | <0.001 |
| 7-class | 41 | 1602821.004 | 1603277.607 | 1603147.307 | 0.77 | 0.1039 | 1 |
| *Abbreviations:* AIC= Akaike Information Criteria; BIC= Bayesian Information Criteria; ssaBIC, sample-size adjusted BIC; LMRT, Lo-Mendell-Rubin Tes; BLRT, bootstrapped likelihood ratio test. | | | | | | | |

**Supplementary Figure S2.** Estimated indicator probabilities and latent classes for all five dimensions of SDQ in children (*N* = 389,125).

*Note:* These probabilities correspond to the dichotomized response scale (0 = Normal/Borderline; 1 = Abnormal), where higher values indicate greater likelihood of exhibiting problems in each respective dimension.

*Abbreviations:* SDQ, Strengths and Difficulties Questionnaire; Class 1, Low symptom; Class 2, Internalizing; Class 3, Peer and prosocial issues; Class 4, High difficulties; Class 5, Hyperactive.

**Supplementary Figure S3.** Estimated indicator probabilities and latent classes for all five dimensions of SDQ in adolescents (*N* = 118,063).

*Note:* These probabilities correspond to the dichotomized response scale (0 = Normal/Borderline; 1 = Abnormal), where higher values indicate greater likelihood of exhibiting problems in each respective dimension.

*Abbreviations:* SDQ, Strengths and Difficulties Questionnaire; Class 1, Low symptom; Class 2, Internalizing; Class 3, Peer and prosocial issues; Class 4, High difficulties; Class 5, Hyperactive.
